# Retrospective Evaluation of Recombinant Human Brain Natriuretic Peptide Therapy for Decompensated Right Heart Failure Across Pulmonary Hypertension Groups

**DOI:** 10.3390/medicina62071213

**Published:** 2026-06-23

**Authors:** Lixing Hu, Qing Zhao, Zhihui Zhao, Qin Luo, Li Deng, Zhihong Liu

**Affiliations:** Center for Respiratory and Pulmonary Vascular Diseases, Department of Cardiology, Fuwai Hospital, National Clinical Research Center for Cardiovascular Diseases, National Center for Cardiovascular Diseases, Chinese Academy of Medical Sciences and Peking Union Medical College, Beijing 100037, China; hulx08@126.com (L.H.); fuwailiu@hotmail.com (Z.Z.); luoqin2009@163.com (Q.L.); dengdengchristina@163.com (L.D.)

**Keywords:** pulmonary hypertension, recombinant human brain natriuretic peptide, heart failure, efficacy, safety

## Abstract

*Background and Objectives*: Right heart failure is a life-threatening complication of pulmonary hypertension (PH), with limited treatment options. Although recombinant human brain natriuretic peptide (rhBNP) is widely used in left heart failure, its effectiveness in right heart failure associated with varying groups of PH (Groups 1, 2, and 4) is unknown. *Materials and Methods*: 763 patients with varying groups of PH (PH Groups 1, 2, and 4) were enrolled and received both conventional therapy and rhBNP treatment. Therapeutic efficacy and adverse event incidence were evaluated among the PH groups. *Results*: Significant reductions in variables reflecting cardiac congestion, including NT-proBNP, total bilirubin, and body weight, were observed in all PH subgroups (all *p* < 0.001). The median percentage changes were −47% (IQR −76 to −24), −21% (IQR −33 to −1), and −3% (IQR −7 to −1), respectively. Alanine transaminase levels presented a decreasing trend (*p* < 0.001), whereas creatinine levels remained unchanged (*p* > 0.05), with consistent trends across PH subgroups. The hemodynamic response was heterogeneous, with marked decreases in the mean arterial pressure in Groups 1 and 4 (*p* < 0.001) but not in Group 2. Improvement in dyspnea and edema of the lower limbs was observed in 49.9% and 66.6% of cases, respectively. The overall incidence of adverse events was 0.66%, with 0.26% (2/763) being serious, all of which were in Group 1 PH. *Conclusions*: Findings from this exploratory analysis indicated that rhBNP treatment was associated with favorable changes in congestive status and clinical symptoms across different PH subgroups, as well as stable end-organ function. Of note, all patients received comprehensive conventional background therapy; thus, these improvements cannot be exclusively attributed to rhBNP alone. Given the observed hemodynamic fluctuations, close blood pressure monitoring should be considered throughout the treatment course, particularly for patients in Groups 1 and 4, and most notably for high-risk PAH patients (Group 1 PH).

## 1. Introduction

Pulmonary hypertension (PH) is defined by the presence of abnormally raised pressure in the pulmonary arteries, adversely affecting right ventricle (RV) hemodynamics [1]. Over time, this increased afterload leads to RV strain, dysfunction, and ultimately right heart failure, associated with high degrees of morbidity and mortality [2]. Acute decompensated right heart failure requires careful decongestion, often with diuretics. However, overaggressive diuresis may exacerbate renal dysfunction and adversely impact clinical outcomes [2]. It is thus important to effectively reduce the load on the heart while preserving end-organ function.

Brain natriuretic peptide (BNP) is produced by ventricular myocardial tissue under conditions of pressure and volume overload [3]. It promotes natriuresis, vasodilation, and inhibits the renin–angiotensin–aldosterone system, thereby counteracting the volume overload and pathological remodeling associated with heart failure [4]. Recombinant human BNP (rhBNP), a synthetic form identical to endogenous BNP, received approval from the U.S. Food and Drug Administration in 2001 for treating acute decompensated left heart failure [5]. Beyond its decongestive properties, rhBNP can reduce ventricular filling pressure, attenuate myocardial fibrosis, inhibit adverse remodeling, and modulate inflammatory responses, without compromising renal function [5,6,7,8].

rhBNP has been shown to benefit hemodynamics in patients with post-capillary PH (representing Group 2 PH), including significant reductions in pulmonary arterial pressure and vascular resistance, as well as biventricular filling pressure [7,9,10,11]. Moreover, emerging evidence suggests that rhBNP may also be effective in treating non-Group 2 forms of PH. For instance, in cases of RV dysfunction following acute pulmonary embolism, administration of rhBNP improved RV function and reduced rehospitalization rates [12]. Its effects may be partly mediated by a greater bioavailability of nitric oxide (NO), which modulates pulmonary vascular tone and attenuates hypoxic vasoconstriction [13,14].

Despite these promising findings, the application of rhBNP in right heart failure, especially in non-Group 2 PH, including pulmonary arterial hypertension (PAH) and chronic thromboembolic pulmonary hypertension (CTEPH), remains underexplored, and its safety and efficacy in different PH subgroups have not been systematically evaluated. Specifically, there has been no direct comparison of the therapeutic profiles of rhBNP between cases of Group 2 PH and non-Group 2 PH (i.e., PAH and CTEPH).

Therefore, this retrospective, single-arm, observational study aimed to preliminarily explore the efficacy and safety of rhBNP in patients with acute decompensated right heart failure across different pulmonary hypertension subgroups (Groups 1, 2, and 4), with a specific focus on preliminary comparison of outcomes among these groups. Patients with Group 3 PH (due to lung disease/hypoxia) and Group 5 PH (unclear/multifactorial mechanisms) were not included in this study. Therefore, our findings are applicable only to patients with right heart failure secondary to Groups 1, 2, and 4 PH and should not be extrapolated to other PH subgroups. We sought to provide a foundation for potentially broadening the use of rhBNP in a wider spectrum of pulmonary hypertension and to generate hypothesis-forming evidence to inform future randomized controlled trials.

## 2. Materials and Methods

### 2.1. Patient Profile

This retrospective cohort study enrolled patients admitted to the Respiratory and Pulmonary Vascular Diseases Center at Fuwai Hospital, China, between January 2019 and June 2024. This study followed the guidelines of the Declaration of Helsinki and was approved by the Ethics Committee of Fuwai Hospital (approval number 2022-1713). All participants provided written informed consent.

Inclusion criteria: (1) Diagnosis of PH (Groups 1, 2, or 4) based on the 2018 World Symposium on Pulmonary Hypertension guidelines, with confirmation by right heart catheterization within the preceding five years; (2) hospitalized for acute decompensated right heart failure; (3) World Health Organization (WHO) functional class II–IV; (4) no prior use of rhBNP and no known allergy or contraindication to it; and (5) a level of NT-proBNP of ≥1400 pg/mL.

Exclusion criteria: (1) Pulmonary veno-occlusive disease or pulmonary capillary hemangiomatosis; (2) unstable angina (high-risk) or acute myocardial infarction; (3) significant valvular heart disease (e.g., aortic stenosis); (4) acute aortic dissection, acute pulmonary embolism, or pulmonary infarction; (5) acute pericarditis, myocarditis, or restrictive cardiomyopathy; (6) thrombophlebitis or intracardiac thrombus; (7) second- or third-degree atrioventricular block or severe supraventricular/ventricular arrhythmia; (8) acute stroke; and (9) severe hepatic or renal dysfunction or severe hypotension.

Classification of pulmonary hypertension groups. Participants were divided into three groups in terms of clinical and hemodynamic criteria. These included Group 1 (PAH): Presence of right heart failure resulting from PAH, represented by a mean pulmonary arterial pressure (mPAP) of ≥25 mmHg, a pulmonary capillary wedge pressure of (PCWP) ≤ 15 mmHg, and a pulmonary vascular resistance of (PVR) > 3 Wood units; Group 2 (PH due to left-sided heart disease): Presence of right heart failure resulting from left-sided heart disease, with a mPAP ≥ 25 mmHg and PCWP > 15 mmHg; Group 4 (CTEPH): Presence of right heart failure caused by CTEPH, with a mPAP ≥ 25 mmHg and PVR > 3 Wood units.

### 2.2. Treatment Procedures

All patients received conventional background therapy and standard care, including diuretics, oxygen, inotropes/vasoactives, targeted PH therapies, bed rest, and dietary sodium/fluid control. Diuretics were generally administered at low doses as follows: furosemide 20–40 mg, torasemide 20–40 mg, or bumetanide 1 mg intravenously once daily. Spironolactone was given at 20 mg once daily. Tolvaptan was typically prescribed at 7.5 mg once daily, adjusted based on serum sodium levels; it was discontinued if the serum sodium level exceeded 144 mmol/L. Thiazide diuretics were essentially not used. After evaluation by physicians, targeted drug therapy was given according to the 2018 guidelines of the World Symposium on Pulmonary Hypertension [15]. Sildenafil was administered at 20 mg three times daily; tadalafil at 20–40 mg once daily; ambrisentan at 5–10 mg once daily; and macitentan at 10 mg once daily. Riociguat was initiated at 1 mg three times daily, with gradual dose escalation up to a maximum single dose of 2.5 mg three times daily, based on systolic blood pressure and tolerability. Treprostinil was initiated at 1.25 ng/kg/min via intravenous or subcutaneous route, with gradual dose escalation guided by systolic blood pressure and tolerability. All patients with chronic thromboembolic pulmonary hypertension (CTEPH) received adequate therapeutic doses of anticoagulation. Intravenous infusion of rhBNP (brand name: XinHuoSu^®^; manufacturer: Chengdu Nordikang Biopharmaceutical Co., Ltd.; Chengdu, China, batch number: gyZZ S20050033) was initiated within 24 h of all eligible admissions. Initial Regimen: All patients received rhBNP via continuous intravenous infusion without a loading dose at a starting maintenance dose of 0.003–0.01 μg/kg/min. Based on our center’s prior clinical experience, high-risk PAH patients are highly sensitive to preload reduction; therefore, a loading dose was intentionally omitted to minimize the risk of hypotension, a common adverse event associated with rhBNP administration. Dose adjustments were strictly based on three predefined parameters, with all adjustments confirmed by the attending physicians to ensure consistency. The specific criteria were as follows: ① Blood Pressure (BP): BP was monitored at baseline, one hour post-initiation, 30–60 min post-adjustment, and every 12 h after stabilization. Hypotension was defined as either symptomatic (dizziness, lightheadedness, or syncope) or asymptomatic [systolic BP (SBP) < 85 mmHg or a >20 mmHg drop from baseline]. Persistent hypotension prompted the addition of vasoactive agents (dopamine or dobutamine 100–300 μg/min, or norepinephrine 1–3 μg/min). If hypotension persisted despite vasoactive support, the rhBNP dose was immediately halved. If the systolic BP further fell below 80 mmHg, the infusion was temporarily paused and resumed at half the previous dose once the systolic BP recovered to a stable level (≥90 mmHg). ② Clinical Response: If patients showed obvious improvement in clinical symptoms (e.g., alleviation of dyspnea, reduction in fatigue) and an increase in urine output, the current rhBNP dose was maintained. If symptom improvement was inadequate while their blood pressure remained stable (SBP ≥ 90 mmHg), the dose could be increased by 0.003–0.004 μg/kg/min every 12 h, with a maximum dose not exceeding 0.01 μg/kg/min. ③ Weight Change: If a patient’s body weight decreased excessively (>1.5 kg/day), the rhBNP dose was reduced or paused appropriately to avoid excessive preload reduction, which could lead to hemodynamic instability. Due to reimbursement policies that limit rhBNP administration to a maximum of 3 consecutive days, the infusion duration was highly standardized across all tolerant patients.

### 2.3. Data Collection

A standardized protocol was implemented for systematic data collection from the study participants. Comprehensive medical histories were documented at baseline, with particular focus placed on right heart failure symptoms and signs (such as dyspnea and peripheral edema), the WHO’s functional class, and records of whether medications were used (diuretics, inotropes/vasoactives). Due to the retrospective nature of this study, detailed data on changes in concomitant medication doses (including diuretics, inotropes/vasoactives, and PH-targeted therapies) during the rhBNP infusion period were not systematically captured and are therefore unavailable. Vital signs, such as heart rate, blood pressure, and daily fluid balance (24 h intake, output, body weight), were monitored rigorously throughout hospitalization. Dyspnea improvement was defined as patient-reported “significant improvement or complete relief” or physician-documented “dyspnea improved or increased exercise tolerance,” and was assessed independently by two researchers who were both attending physicians in cardiology (with discrepancies resolved by a third researcher). Fasting venous blood samples were analyzed for NT-proBNP, total bilirubin (TBil), indicators of liver and kidney function, and electrolyte levels before and after rhBNP infusion. Transthoracic echocardiography was conducted at the same time points to assess cardiac structure and function, measuring the left ventricular end-diastolic diameter (LVEDD), right ventricular end-diastolic diameter (RVEDD), and tricuspid annular plane systolic excursion (TAPSE). Post-treatment parameters were obtained according to the following pre-specified rules: for patients hospitalized >3 days, parameters were collected on Day 4 (i.e., the day after completing the 3-day rhBNP infusion). For patients discharged early (≤3 days), parameters were collected at the time of discharge. All adverse events, such as symptomatic hypotension, arrhythmia, cardiogenic shock, sudden cardiac-related and all-cause death during hospitalization, were documented. All participants underwent follow-up until 30 days after discharge to capture complete clinical outcomes.

### 2.4. Outcome Definitions

The primary endpoint was defined as the change in NT-proBNP levels before and after rhBNP treatment. Secondary endpoints included alterations in 24 h fluid intake, urine output, body weight, hemodynamic indicators, liver and renal function, electrolytes, echocardiographic parameters, and clinical symptom improvements.

### 2.5. Statistical Analysis

Data were analyzed using SPSS version 26 (IBM Corp., Armonk, NY, USA) and DMSAS Version 1.0 (Guangzhou Bowei Data Technology Co., Ltd., Guangzhou, China. https://www.dmsas.cn) (accessed on 20 January 2026). Graphs were compiled using GraphPad Prism version 9.0 software (GraphPad Software, San Diego, CA, USA). Normally distributed continuous variables are presented as mean ± standard deviation, while those with non-normal distributions are shown as the median with an interquartile range (M [IQR]). Categorical variables are expressed as absolute numbers and percentages. Longitudinal changes in daily intake, urine output, and body weight from baseline were analyzed using a mixed-effects model with random intercepts, time as a fixed effect, and robust standard errors by DMSAS. Within-group comparisons of pre- and post-treatment measurements were conducted using paired *t*-tests for variables with normal distributions, and Wilcoxon signed-rank tests for non-normally distributed variables. Differences in the rates of dyspnea and edema improvement were assessed using chi-square tests. Percentage changes for continuous variables were determined as: (post-treatment value − pre-treatment value)/pre-treatment value × 100%. These percentage changes were then used for inter-group comparisons. One-way ANOVAs were utilized for normally distributed percentage changes, and Kruskal–Wallis H tests were applied for those with skewed distributions. For NT-proBNP and TBil, which exhibited markedly skewed distributions, a natural logarithmic transformation (ln) was applied to satisfy the normality assumption. An analysis of covariance (ANCOVA) was performed to compare treatment responses among groups, with the post-treatment ln(NT-proBNP) and ln(TBil) as the dependent variables, treatment group as the fixed factor, and the pre-treatment ln(NT-proBNP) and ln(TBil) as the covariates, respectively. If the global test indicated a significant difference (*p* < 0.05), pre-specified pairwise comparisons (Group 1 vs. Group 2 and Group 4 vs. Group 2) were conducted using *t*-tests (normal distribution) or Mann–Whitney U tests (non-normal distribution), with application of the Bonferroni correction for multiple comparisons. To further adjust for potential confounders, a multivariate regression model was conducted with the change in ln(NT-proBNP) and ln(TBil) as the dependent variables, and covariates, including age, sex, comorbidities, baseline ln(NT-proBNP)/ln(TBil), use of diuretics, use of inotropes/vasoactives, baseline creatinine level, and WHO functional class. A two-sided *p*-value < 0.025 was considered statistically significant for these inter-group comparisons, while *p* < 0.05 represented significance in all other tests.

## 3. Results

### 3.1. Baseline Features and Volume Management

A consecutive series of 851 eligible patients was enrolled over a 5.5-year period. After excluding patients with concurrent Group 1 PH combined with Group 2 or Group 4 PH, 763 patients (89.7%) remained for final analysis (288 male, 475 female), including 483 (63.3%) with Group 1, 68 (8.9%) with Group 2, and 212 (27.8%) with Group 4 PH. Baseline demographic and clinical information is summarized in Table 1. The average age was 49.62 ± 16.72 years. Compared with those in Group 2 PH, patients in Group 1 were significantly younger, more likely to be female, and had much lower weight (*p* < 0.05). Group 2 participants used fewer inotropes/vasoactives and were more likely to have comorbidities such as hypertension, hyperlipidemia, diabetes mellitus, coronary heart disease, and atrial fibrillation/flutter relative to those in Groups 1 and 4 (all *p* < 0.05). The median hospitalization duration differed significantly across groups: overall 7 (6–11) days, with Group 1 at 7 (5–10) days, Group 2 at 9 (6–14) days, and Group 4 at 8 (6–11) days. Notably, Group 1 had the shortest stay.

**Figure 1:**
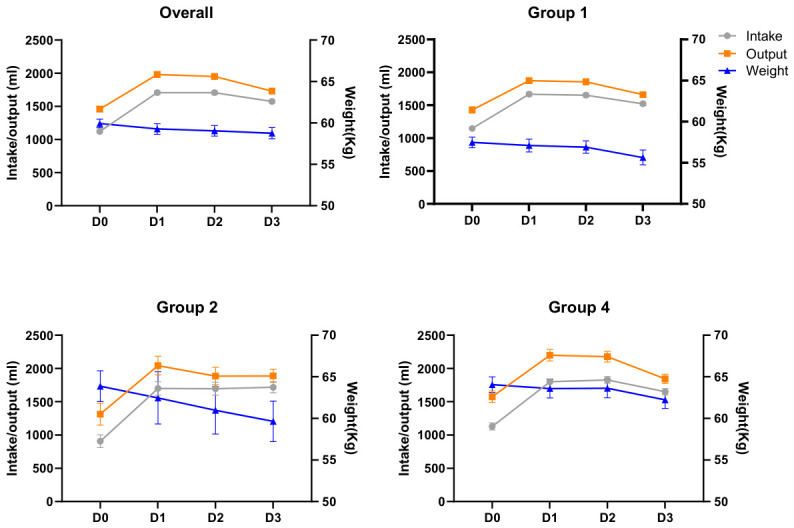
Daily volume status of 24 h intake, urine output, and body weight (kg) changes.

Volume status improvement was observed following rhBNP administration across all PH groups. As detailed in Table 2 and Figure 1, marked increases in 24 h fluid intake and output, accompanied by significantly reduced body weights, were observed on the first, second, and third days of treatment relative to baseline (all *p* < 0.05).

### 3.2. Cardiac Congestion Parameters and Hemodynamics

In the overall PH cohort, significant reductions in NT-proBNP, total bilirubin, and body weight levels were observed: NT-proBNP decreased from 2706 (IQR 1687–4632) pg/mL to 1432 (IQR 714–2679) pg/mL (*p* < 0.001), total bilirubin from 21 (IQR 14–34) µmol/L to 18 (IQR 12–29) µmol/L (*p* < 0.001), and body weight from 61.4 ± 14.0 kg to 58.7 ± 13.0 kg (*p* < 0.001) (Table 3). The corresponding median percentage changes were −45%, −20%, and −3%, respectively.

**Figure 2:**
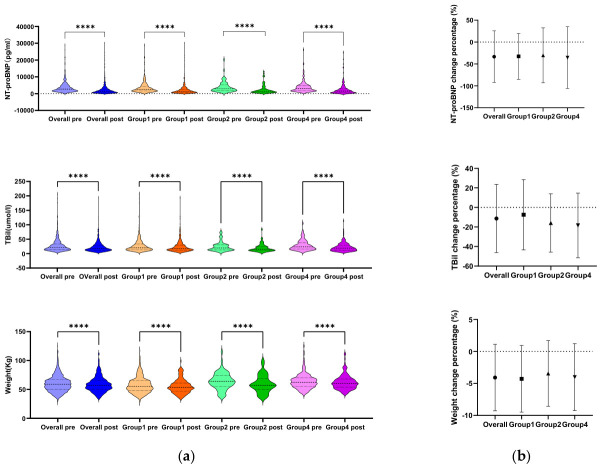
(**a**) The NT-proBNP, TBil, and weight levels before and after rhBNP treatment; (**b**) the change percentage of NT-proBNP, TBil, and weight. ****: *p* < 0.001 for comparison between pre- and post-rhBNP.

Consistent improvements in these parameters were observed in all PH subgroups. In Group 1, NT-proBNP levels decreased from 2590 (1591–4359) pg/mL to 1383 (724–2626) pg/mL, with a median percentage change of −43%, while total bilirubin decreased from 21 (14–33) µmol/L to 18 (12–29) µmol/L (−20%), and weight from 58 ± 13 kg to 56 ± 12 kg (−4%). In Group 2, the values were 2879 (1826–5259) pg/mL to 1436 (788–2678) pg/mL (−26%) for NT-proBNP, 20 (13–31) µmol/L to 15 (11–24) µmol/L (−21%) for total bilirubin, and 62 ± 17 kg to 60 ± 15 kg (−6%) for body weight. In Group 4, the values were 3131 (1792–4831) pg/mL to 1522 (609–2910) pg/mL (−47%) for NT-proBNP, 24 (16–38) µmol/L to 19 (12–29) µmol/L (−21%) for total bilirubin, and 65 ± 14 kg to 62 ± 12 kg (−3%) for body weight. All within-group shifts were significant (*p* < 0.001) (Figure 2).

The hemodynamic responses varied among the subgroups (Table 3). While the mean arterial pressure (MAP) values were found to decrease markedly overall and in Groups 1 and 4 (all *p* < 0.001), there was no substantial change in the Group 2 participants. The heart rate values were markedly reduced both overall and in Group 1 (*p* < 0.001) but remained unchanged in Groups 2 and 4.

### 3.3. Impact on Organ Function, Electrolyte Levels, and Echocardiographic Parameters

Improvements in hepatic and metabolic profiles were observed in all PH groups following rhBNP administration. Alanine transaminase (ALT), uric acid (UA), serum sodium (Na+), and potassium (K+) level reductions were observed in the overall cohort (*p* < 0.001) (Table 3). Baseline creatinine (Cr) and eGFR data are presented in Table 3. No marked changes in Cr and eGFR levels were observed following treatment, and no acute kidney injury was noted in association with rhBNP use in this cohort. These trends were largely consistent across the PH subgroups (Table 3).

The echocardiographic parameters remained largely stable. A marked but modest reduction in RVEDD was observed in both the overall population and in Group 2 (*p* < 0.05), while the TAPSE and LVEDD values were not altered significantly in any of the groups (Table 3).

### 3.4. Comparison of Parameters Among Participants with Group 2 Versus Group 1 or 4 PH

A comparative analysis revealed no significant differences in the percentage changes in most parameters, including those associated with cardiac congestion, renal and liver function, electrolyte levels, echocardiographic measures (TAPSE, LVEDD), and hemodynamic variables, between Group 2 and Groups 1 or 4 (all *p* > 0.025). An exception to this trend was observed in RVEDD values, which showed a greater reduction in Group 2 compared to Group 1 (median percentage change: 0% [IQR 0 to 0] vs. 0% [IQR −5 to 0], *p* < 0.025). No marked changes in RVEDD values were seen between Groups 2 and 4 (*p* > 0.025) (Table 3). After ln-transformation, ANCOVA with baseline ln(NT-proBNP) as a covariate showed no significant post-treatment differences between Group 1 vs. Group 2 (*p* = 0.324) or Group 2 vs. Group 4 (*p* = 0.337). Multivariate regression, adjusting for age, sex, comorbidities, baseline ln(NT-proBNP), diuretics, inotropes/vasoactives, baseline creatinine, complications, and WHO class, confirmed non-significant differences for both comparisons (*p* = 0.547 and *p* = 0.842, respectively). After ln-transformation, ANCOVA with baseline ln(TBil) as a covariate showed no significant post-treatment differences between Group 1 vs. Group 2 (*p* = 0.034) or Group 2 vs. Group 4 (*p* = 0.817). Multivariate regression, adjusting for age, sex, comorbidities, baseline ln(TBil), diuretics, inotropes/vasoactives, baseline creatinine, complications, and WHO class, confirmed non-significant differences for both comparisons (*p* = 0.420 and *p* = 0.408, respectively).

### 3.5. Symptomatic Improvements and Safety Outcomes

**Figure 3:**
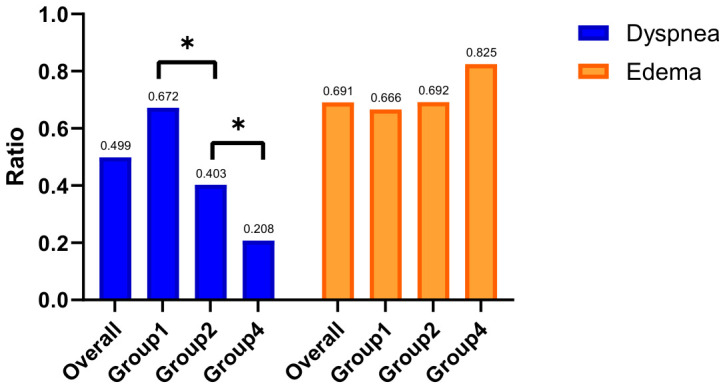
The comparison of the improvement rate of dyspnea and lower-extremity edema. * *p* < 0.025 Chi-square test.

After an analysis of changes in symptoms after treatment, substantial differences in the alleviation of dyspnea among the PH groups (*p* < 0.001) were observed, with the highest rate of alleviation seen in Group 1 (67.2%), an intermediate rate in Group 2 (40.3%), and the lowest in Group 4 (20.8%). Post hoc comparisons confirmed marked differences between Groups 1 and 2 (x2 = 17.63, *p* < 0.001) and Groups 4 and 2 (x2 = 9.927, *p* = 0.002). Conversely, improvements in lower-extremity edema were consistently high in all groups, with no significant variations among the groups (all *p* > 0.05). Of the 521 patients with lower-extremity edema, 360 showed improvement, corresponding to an overall improvement rate of 69.1%. Among the 428 participants with Group 1 PH and lower-extremity edema, 285 showed improvement (66.6%). Similarly, improvements were noted in 9 of the 13 patients with Group 2 PH (69.2%), and in 66 of the 80 participants with Group 4 PH (82.5%) (Figure 3).

The overall incidence of adverse events was 0.66% (5/763), including three non-serious symptomatic hypotension cases (0.39%) that resolved within 15 min after fluid resuscitation or vasoactive agents, and two serious adverse events (0.26%) resulting in irreversible unconsciousness and fatal outcomes. Both deaths occurred in patients classified as high-risk pulmonary hypertension. Case 1: A 38-year-old male with congenital heart disease-associated PH. Approximately 40 h after rhBNP initiation (0.0067 μg/kg/min), he suddenly collapsed; his blood pressure dropped from 100/85 mmHg to 56/18 mmHg, followed by respiratory and cardiac arrest. Concomitant medications included diuretics, digoxin, and levosimendan. Case 2: A 51-year-old female with connective tissue disease-associated PH and a history of recurrent syncope. After >20 h of rhBNP infusion (0.0067 μg/kg/min), she developed ventricular fibrillation and died despite resuscitation. Concomitant medications included prednisolone, hydroxychloroquine, macitentan, selexipag, tadalafil, rivaroxaban, spironolactone, bumetanide, calcitriol, levothyroxine, potassium chloride, and metoprolol sustained-release (tablet form). In both cases, no rhBNP dose adjustment was made prior to the events. All recorded adverse events were observed in patients with PAH. Three cases of asymptomatic hypotension were detected during routine monitoring. Blood pressure normalized following discontinuation of rhBNP, and these occurrences were not classified as adverse events.

### 3.6. 30-Day Follow-Up Outcomes

Clinically meaningful follow-up outcomes, including 30-day mortality, in-hospital therapy escalation, and 30-day readmission, were assessed. Within 30 days, five deaths occurred (four in Group 1, one in Group 4). Beyond the two cases detailed above, the remaining three deaths were attributed to progressive right heart failure. In-hospital therapy escalation (i.e., transfer to the intensive care unit) was required for the two fatal cases described above. No patients were readmitted within 30 days of discharge.

The text continues here (Figure 2 and Table 2).

## 4. Discussion

Right heart failure is a life-threatening complication of PH, for which there are few treatment options [16]. Although rhBNP therapy is widely used in left heart failure [5], there is limited information on its effectiveness in treating right heart failure associated with different PH subgroups. This observational study preliminarily explored the efficacy and safety of rhBNP in patients with acute decompensated right heart failure across different pulmonary hypertension subgroups (Groups 1, 2, and 4), with the intention to provide a foundation for potentially broadening the use of rhBNP in a wider spectrum of pulmonary hypertension and to generate hypothesis-forming evidence to inform future randomized controlled trials.

Markedly increased 24 h fluid intake and output, along with reduced body weight, were observed in association with rhBNP administration. This is consistent with our earlier findings regarding the association of rhBNP with outcomes in individuals with Group 1 PH [17]. Meanwhile, the median 3% reduction in body weight (approximately 1.8 kg over three days) observed in this study is modest compared to conventional decongestion targets of 1–1.5 kg per day. Because patients with pulmonary hypertension and right ventricular dysfunction are typically preload-dependent, aggressive decongestion may precipitate a significant drop in preload, leading to decreased cardiac output, systemic hypotension, and even fatal arrhythmias—an adverse event profile noted during this study. Therefore, a more gradual diuresis strategy was intentionally adopted to maintain hemodynamic stability. The modest weight reduction reflects a conservative decongestion approach aimed at balancing fluid removal with right ventricular preload preservation. Reduced NT-proBNP levels, along with alleviation of the clinical signs and symptoms of heart failure, were also observed in association with rhBNP administration. NT-proBNP is a key indicator used in assessing the severity of heart failure [16]. Reduced levels of this indicator suggest that the addition of rhBNP administration to conventional treatment is associated with the alleviation of symptoms and mitigation of PH-related right-sided heart failure to a degree. Improvements in indicators of hepatic congestion, specifically in the levels of transaminase and bilirubin, were also observed following rhBNP treatment. Hepatic congestion and reduced hepatic perfusion can induce cirrhosis, and hyperbilirubinemia is known to be associated with a poor prognosis of PH [18]. No marked changes were seen in Cr levels, indicating no renal compromise, suggesting that rhBNP may also associate with preservation of end-organ function. However, it should be noted that patients with severe renal dysfunction were excluded, and baseline renal function was generally preserved. Therefore, stable serum creatinine does not constitute robust evidence for renoprotection. Uric acid is an important marker that reflects disease progression and treatment response in individuals with PAH-associated right heart failure [19]. A meta-analysis showed that raised levels of serum uric acid are linked to a greater likelihood of adverse outcomes in patients with heart failure [20]. And our previous study showed that baseline hyperuricemia was related to a higher rate of 5-year mortality in patients with idiopathic pulmonary arterial hypertension (IPAH) [21]. Here, marked decreases in uric acid levels were observed following rhBNP administration. These findings suggested that rhBNP administration might be associated with alleviation of clinical symptoms and right heart failure-related conditions, as well as preservation of end-organ function, and might also be associated with improved prognosis in various groups of PH. However, the observed improvements in NT-proBNP, urine output, body weight, dyspnea, and edema could not be definitively separated from the effects of standard inpatient care (including diuretics, oxygen, inotropes/vasoactives, targeted therapy adjustments, bed rest, dietary sodium/fluid control, etc.).

The observed reduction in serum sodium (*p* < 0.001) also requires reconciliation with the natriuretic properties of BNP, as traditional understanding suggests BNP promotes natriuresis and should maintain or reduce sodium retention. But in the context of hemodynamic instability or renal impairment, the diuretic and natriuretic effects of BNP may be diminished, potentially leading to sodium retention despite pharmacological intervention. Although statistically significant, the absolute magnitude of serum sodium reduction was modest, asymptomatic, and may have little clinical relevance; a similar pattern was also observed for serum potassium.

Interestingly, the echocardiographic indices in this cohort remained relatively stable after rhBNP treatment. Although an exception was observed in RVEDD, the change was relatively small and may have little clinical significance. Several factors may explain this observation: the relatively short duration of treatment may have been insufficient to enable detection of reverse remodeling, and the primary effects of rhBNP may be on the alleviation of symptoms and congestion rather than direct modifications of the heart structure within the short period.

Importantly, when comparing Group 2 PH with non-*G*roup 2 PH (Groups 1 and 4), no marked differences were found in key endpoints, including changes in NT-proBNP, weight, hepatic/renal function, and electrolyte levels. These results indicated that rhBNP, when added to conventional diuretic therapy, is associated with effective decongestion and end-organ preservation in non-Group 2 PH, with comparable endpoints to those seen in Group 2 PH, while the PH groups differed substantially in age, sex distribution, body weight, comorbidities, inotropes/vasoactives use, and hospitalization duration. We performed adjusted analyses for NT-proBNP and TBil, which may provide some mitigation of confounding. However, many other outcomes, including symptom improvement, hemodynamic changes, and adverse events, remain vulnerable to confounding and should therefore be interpreted with caution. Groups 1 and 4 PH fall into the category of pre-capillary PH, and their treatment principles differ significantly from those of Group 2 PH. There is insufficient evidence to support the use of rhBNP in treating Groups 1 and 4 PH-associated right heart failure. It has been found that in cases of right-sided heart failure resulting from pre- or post-capillary PH, rhBNP administration induces increases in NO and cyclic guanosine monophosphate (cGMP) [13]. Patients with primary and secondary PH show marked decreases in the levels of vasodilator NO (NOx) [13], while plasma and urinary cGMP/NO levels, which serve as direct readouts of NPRA (Natriuretic Peptide Receptor A) activation, were not measured in this study, as these assays are not currently available at our center. Therefore, theoretically speaking, rhBNP therapy may reduce arterial pressure and capillary resistance in the lungs by increasing the concentrations of both NO and cGMP, thereby reducing the symptoms of right-sided heart failure. Future studies incorporating cGMP/NO monitoring are warranted to further elucidate the mechanistic effects of rhBNP in pulmonary hypertension.

Another notable finding in this study was the dissociation between symptom improvement and biochemical or hemodynamic changes. Group 1 patients demonstrated higher rates of dyspnea improvement, and experienced significant mean arterial pressure reductions yet only comparable NT-proBNP reductions to Group patients. Several potential explanations for this discrepancy warrant consideration. First, the pathophysiological mechanisms are substantially distinct. Left heart failure-related PH is dominated by pulmonary congestion, whereas Group 1 pulmonary hypertension is more closely linked to exercise tolerance and exertional symptoms. During hospitalization, strict bed rest and restricted physical activity largely minimize exertional burden for patients with Group 1 PH, which may independently alleviate perceived dyspnea and further weaken the correlation between subjective symptoms and objective hemodynamic or biomarker changes. Second, Group 1 patients were generally younger and possessed greater physiological reserve, enabling more noticeable symptomatic improvement, even in the setting of modest objective improvements. Furthermore, dyspnea perception in pulmonary hypertension is multifactorial and does not always correlate closely with conventional markers of systemic congestion or hemodynamic status. Moreover, we acknowledge that dyspnea was not assessed via a standardized, validated scale in this retrospective cohort, and no qualified inter-rater agreement was calculated, inevitably introducing more subjectivity to symptomatic evaluations. Future prospective studies should adopt standardized assessment tools, such as the Borg dyspnea scale, and combine patient-reported outcomes with objective biomarkers and qualified inter-rater agreement to achieve a more comprehensive and rigorous evaluation of clinical improvement.

In terms of safety, hypotension was the most frequently observed adverse event. While the MAP was markedly reduced in both Groups 1 and 4 PH, Group 2 PH was not affected. Therefore, blood pressure should be carefully monitored in cases of heart failure treated with rhBNP, especially in those with Groups 1 or 4 PH, and treatment should be reduced or halted if symptomatic hypotension develops. Here, it was found that halting rhBNP treatment led to rapid increases in blood pressure. Renal function was not significantly altered, and no new arrhythmia events were observed. Two high-risk pulmonary hypertension patients died during rhBNP administration. One death might be associated with preload reduction in a volume-sensitive patient; the other was attributed to malignant arrhythmia. These events underscore the importance of close hemodynamic monitoring during rhBNP use, particularly in high-risk patients. However, given the poor prognosis of critically ill PAH patients (a French study reported 41% fatality rates of patients with critical pulmonary hypertension in the intensive care unit [22]) and the severity of underlying disease in these two cases, causality cannot be determined from this study design. The significantly lower incidence of adverse events in this study compared with the literature [22] might be explained by the exclusion criteria, which selected a relatively stable study population by excluding patients with severe hypotension and advanced organ dysfunction. Therefore, although a definite causal relationship cannot be established, this safety signal warrants a much more cautious interpretation. As high-risk PAH patients may be particularly vulnerable to preload reduction and hypotension, extrapolation of the safety profile of rhBNP to sicker patients, particularly those with advanced pre-capillary disease, is not straightforward and should be objective.

The study has several limitations. First, as a retrospective, single-arm, real-world study, it lacked a control group, and the observed improvements cannot be definitively attributed to rhBNP. Accordingly, we adopted an associative rather than causal interpretive framework. Second, the exclusion of patients with severe organ dysfunction, hypotension, arrhythmias, and acute pulmonary embolism—precisely the high-risk populations where therapeutic decisions are most challenging—limits generalizability. The single-center design at a specialized pulmonary vascular center further restricts applicability to community settings. Third, invasive hemodynamic data (e.g., right heart catheterization) were not available. While mean arterial pressure and heart rate provide limited information, repeated invasive procedures were not clinically justified in critically ill patients. Future studies may consider pulmonary artery catheterization in carefully selected patients. Fourth, detailed quantitative data on diuretic dosing, inotrope/vasoactive trajectories, and targeted PH therapy adjustments were not systematically captured, limiting our ability to assess potential confounding effects. Additionally, the retrospective design precluded standardized medication documentation and phenotyping (e.g., HFpEF vs. HFrEF in Group 2 PH). Fifth, echocardiographic parameters were limited; additional right ventricular functional measures were generally not obtained due to the critical illness of patients and the use of bedside echocardiography. Sixth, serial biomarker measurements were not obtained at standardized intervals, precluding mixed-effects analysis of temporal trends. The frequency of observation did not permit measurements every 6–12 h, weakening causal inference. Seventh, group sizes were imbalanced (Group 2: n = 68), reducing statistical power for comparisons involving this subgroup; negative findings should be interpreted cautiously due to possible type II errors. Eighth, dyspnea was assessed via patient report and physician notes rather than standardized scales (e.g., Borg), carrying inherent subjective limitations. Ninth, no qualified inter-rater agreement was calculated for the assessment of symptomatic outcomes (e.g., dyspnea), which could compromise the validity and reproducibility of the symptomatic outcome data, particularly when interpreting differences in symptom improvements across PH subgroups. Finally, the follow-up duration was short, and infusion duration was highly standardized due to reimbursement policies (limited to three consecutive days), precluding dose–response analysis. Future prospective, randomized controlled trials with larger sample sizes, standardized medication documentation, comprehensive hemodynamic and echocardiographic endpoints, longer follow-up, and inclusion of high-risk populations are warranted to validate our findings and should incorporate a formal assessment of inter-rater agreement for symptomatic outcomes to enhance data reliability.

## 5. Conclusions

In summary, rhBNP treatment may be associated with favorable improvements in congestion and clinical symptoms, alongside stable end-organ function and a generally acceptable short-term safety profile in patients with right heart failure of varying PH subgroups. Of note, all patients received comprehensive conventional background therapy; thus, these improvements cannot be exclusively attributed to rhBNP alone. In addition, group sizes were imbalanced (Group 2: n = 68), so the interpretation of “no significant differences” between groups should be more cautious. Notably, the two serious adverse events were fatal, although causality cannot be definitively determined due to the lack of a control group; these events are clinically important. High-risk PAH patients (Group 1 PH) may be particularly vulnerable to preload reduction and hypotension associated with rhBNP infusion. Close monitoring of blood pressure and hemodynamics is strongly advised, particularly among individuals in Groups 1 and 4, and most notably for high-risk PAH patients (Group 1 PH). Further prospective controlled studies are warranted to validate these exploratory findings, including the safety profile of rhBNP, and to optimize dosing and administration strategies, and clarify the most appropriate target population for rhBNP in this clinical context.

## Figures and Tables

**Table 1:** Baseline demographics and clinical characteristics of different groups of pulmonary hypertension.

	All Patients (n = 763)	Group 1 (n = 483)	Group 2 (n = 68)	Group 4 (n = 212)
Demographic characteristics Age (years)	49.62 ± 16.72	43.11 ± 14.53 *	62.87 ± 13.56	60.2 ± 14.25
Male, n (%)	288 (37.7%)	144 (29.8%) *	34 (50%)	110 (51.9%)
Weight (Kg)	59.91 ± 13.82	57.48 ± 13.51 *	64.40 ± 14.61	64.04 ± 12.98
WHO function class				
Class II, n (%)	55 (7.2%)	32 (6.6%)	8 (11.8%)	15 (7.1%)
Class III, n (%)	658 (86.2%)	425 (88%)	52 (76.5%)	181 (85.4%)
Class IV, n (%)	50 (6.6%)	26 (5.4%)	8 (11.8%)	16 (7.5%)
Diuretics				
Loop diuretics, n (%)	687 (90%)	434 (89.9%)	61 (89.7%)	192 (90.6%)
Spironolactone, n (%)	723 (94.8%)	462 (95.7%)	61 (89.7%)	200 (94.3%)
tolvaptan, n (%)	309 (40.5%)	181 (37.5%)	29 (42.6%)	99 (46.7%)
Inotropes/vasoactives				
Dopamine, n (%)	64 (8.4%)	34 (7.0%) *	17 (25%)	13 (6.1%) *
Dobutamine, n (%)	224 (29.4%)	154 (31.9%) *	3 (4.4%)	67 (31.6%) *
Norepinephrine, n (%)	16 (2.1%)	15 (3.1%)	0 (0.0%)	1 (0.5%)
Levosimendan, n (%)	167 (21.9%)	100 (20.7%) *	5 (7.4%)	62 (29.2%) *
Complications				
Hypertension, n (%)	171 (22.4%)	66 (13.7%) *	35 (51.5%)	70 (33%) *
Diabetes Mellitus, n (%)	128 (16.8%)	55 (11.4%) *	27 (39.7%)	46 (21.7%) *
Hyperlipidemia, n (%)	143 (18.7%)	54 (11.2%) *	27 (39.7%)	62 (29.2%)
Coronary artery disease, n (%)	67 (8.8%)	16 (3.3%) *	22 (32.4%)	29 (13.7%) *
AF/AFL, n (%)	244 (32.0%)	144 (29.8%) *	39 (57.4%)	61 (28.8%) *
Clinical outcomes				
Days in hospital (day)	7 (6–11)	7 (5–10) *	9 (6–14)	8 (6–11)
Hospital Mortality, n (%)	2 (0.26%)	2 (0.4%)	0 (0%)	0 (0%)
BNP	417 (252–727)	371 (239–659) *	551 (312–1039)	473 (261–777)

Group 1: Group 1 pulmonary hypertension; Group 2: Group 2 pulmonary hypertension; Group 4: Group 4 pulmonary hypertension; AF/AFL: Atrial fibrillation/Atrial flutter; WHO: World Health Organization. * Compared with Group 2 pulmonary hypertension, *p* < 0.05.

**Table 2:** Daily volume status of 24 h intake, 24 h urine output, and body weight.

Parameters	All patients			
	Before rhBNP	First day after rhBNP	Second day after rhBNP	Third day after rhBNP
24 h intake (mL)	1121 ± 718 (n = 622)	1707 ± 677 * (n = 668)	1706 ± 660 * (n = 646)	1574 ± 601 * (n = 666)
24 h UO (mL)	1459 ± 1033 (n = 622)	1979 ± 1018 * (n = 668)	1950 ± 978 * (n = 646)	1731 ± 792 * (n = 666)
Weight (Kg)	59.93 ± 14.00 (n = 708)	59.27 ± 14.17 * (n = 477)	59.05 ± 14.06 * (n = 491)	58.76 ± 13.01 * (n = 373)
Parameters	Group 1 pulmonary hypertension			
	Before rhBNP	First day after rhBNP	Second day after rhBNP	Third day after rhBNP
24 h intake (mL)	1147 ± 726 (n = 395)	1667 ± 667 * (n = 423)	1652 ± 635 * (n = 400)	1521 ± 580 * (n = 425)
24 h UO (mL)	1427 ± 961 (n = 395)	1873 ± 920 * (n = 423)	1854 ± 901 * (n = 400)	1659 ± 720 * (n = 425)
Weight (Kg)	57.49 ± 13.69 (n = 443)	57.10 ± 13.82 * (n = 312)	56.90 ± 13.59 * (n = 321)	55.64 ± 12.13 * (n = 181)
Parameters	Group 2 pulmonary hypertension			
	Before rhBNP	First day after rhBNP	Second day after rhBNP	Third day after rhBNP
24 h intake (mL)	907 ± 709 (n = 54)	1700 ± 779 * (n = 60)	1696 ± 760 * (n = 61)	1717 ± 650 * (n = 61)
24 h UO (mL)	1315 ± 1208 (n = 54)	2043 ± 1091 * (n = 60)	1887 ± 1010 * (n = 61)	1889 ± 762 * (n = 61)
Weight (Kg)	63.87 ± 14.81 (n = 64)	62.46 ± 17.76 * (n = 32)	60.97 ± 16.38 * (n = 33)	59.64 ± 15.22 * (n = 39)
Parameters	Group 4 pulmonary hypertension			
	Before rhBNP	First day after rhBNP	Second day after rhBNP	Third day after rhBNP
24 h intake (mL)	1129 ± 693 (n = 173)	1800 ± 661 * (n = 185)	1828 ± 667 * (n = 185)	1651 ± 618 * (n = 180)
24 h UO (mL)	1576 ± 1124 (n = 173)	2199 ± 1168 * (n = 185)	2177 ± 1089 * (n = 185)	1845 ± 937 * (n = 180)
Weight (Kg)	64.06 ± 13.16 (n = 201)	63.59 ± 12.96 * (n = 133)	63.62 ± 13.48 * (n = 137)	62.23 ± 12.58 * (n = 153)

rhBNP: recombinant human brain natriuretic peptide; UO: urine output. * *p* < 0.05 for comparison with before rhBNP.

**Table 3:** The comparisons of heart failure, hemodynamic, organ function, and ultrasound parameters before and after rhBNP administration.

Parameters		All Patients				Group 1 Pulmonary Hypertension				Group 2 Pulmonary Hypertension				Group 4 Pulmonary Hypertension			
		N	Before rhBNP	After rhBNP	Change% (%)	N	Before rhBNP	After rhBNP	Change% (%)	N	Before rhBNP	After rhBNP	Change% (%)	N	Before rhBNP	After rhBNP	Change% (%)
HF parameters	NT-proBNP (pg/mL)	759	2706 (1687–4632)	1432 (714–2679) **	−45 (−75 to −20)	479	2590 (1591–4359)	1383 (724–2626) **	−43 (−74 to −9)	68	2879 (1826–5259)	1436 (788–2678) **	−26 (−76 to −20)	212	3131 (1792–4831)	1522 (609–2910) **	−47 (−76 to −24)
	TBil (umol/L)	759	21 (14–34)	18 (12–29) **	−20 (−34 to −2)	479	21 (14–33)	18 (12–29) **	−20 (−37 to −1)	68	20 (13–31)	15 (11–24) **	−21 (−32 to −7)	212	24 (16–38)	19 (12–29) **	−21 (−33 to −1)
	Weight (Kg)	371	61.4 ± 14.0	58.7 ± 13.0 **	−3 (−7 to 0)	181	58 ± 13	56 ± 12 **	−4 (−7 to 0)	39	62 ± 17	60 ± 15 **	−6 (−9 to −1)	151	65 ± 14	62 ± 12 **	−3 (−7 to −1)
Hemodynamic parameters	SBP (mmHg)	428	114 ± 16	108 ± 14 **	−6 (−1 to 1)	228	112 ± 15	105 ± 14 **	−6 (−12 to 0)	43	123 ± 18	117 ± 17	−1 (−6 to 13)	157	116 ± 16	109 ± 13 **	−6 (−13 to 1)
	DBP (mmHg)	428	74 ± 13	68 ± 9 **	−6 (−17 to 3)	228	73 ± 13	67 ± 10 **	−7 (−17 to 0)	43	74 ± 12	71 ± 9	0 (−5 to 14)	157	75 ± 13	69 ± 9 **	−7 (−18 to 4)
	MAP (mmHg)	428	87 ± 13	81 ± 10 **	−7 (−14 to 3)	228	86 ± 12	80 ± 10 **	−7 (−14 to 0)	43	90 ± 12	86 ± 10	5 (−3 to 8)	157	88 ± 13	82 ± 9 **	−8 (−14 to 4)
	HR (beat/min)	459	85 ± 17	83 ± 14 **	−3 (−1 to 9)	251	89 ± 17	86 ± 14 **	−4 (−16 to 9)	44	78 ± 18	75 ± 13	−6 (−24 to 7)	164	82 ± 14	81 ± 12	−3 (−12 to 9)
Liver/kidney function and electrolytic parameters	Alt (U/L)	759	17 (11–25)	14 (9–22) **	−22 (−40 to 0)	479	16 (11–24)	14 (8–21) **	−24 (−40 to −4)	68	17 (12–32)	16 (9–24) *	−15 (−48 to 29)	212	17 (12–15)	14 (9–22) **	−21 (−40 to 0)
	Cr (umol/L)	750	79 (64–96)	78 (64–96)	−2 (−12 to 11)	474	73 (61–90) †	73 (62–91) †	−1 (−11 to 10)	66	88 (68–116)	84 (71–114)	0 (−20 to 21)	210	89 (73–107)	85 (73–103)	4 (−10 to 12)
	eGFR (mL/min/1.73 m^2^)	750	86.80 ± 25.57	87.97 ± 26.21 *	1 (−8 to 10)	474	93 ± 24.55 †	94.31 ± 25.83 †	1 (−7 to 9)	67	72.48 ± 26.89	74.95 ± 27.61	1 (−14 to 13)	210	76.19 ± 21.77	77.71 ± 21.56	2 (−8 to 13)
	UA (umol/L)	760	494 (394–580)	442 (352–540) **	−8 (−23 to 4)	480	487 (392–578)	445 (354–545) **	−8 (−23 to 4)	68	506 (381–590)	423 (330–522) *	0 (−38 to 20)	212	507 (411–587)	431 (343–520) **	−9 (−22 to 4)
	Na (mmol/L)	758	141.34 ± 4.01	139.33 ± 3.60 **	−2 (−3 to 0)	479	141.51 ± 3.77	139.52 ± 3.37 **	−1 (−3 to 1)	68	141.04 ± 4.38	139.11 ± 4.56 *	−1 (−5 to 4)	211	141.06 ± 4.40	138.96 ± 3.75 **	−2 (−4 to 0)
	K (mmol/L)	757	4.19 ± 0.54	4.11 ± 0.41 **	−4 (−10 to 7)	479	4.16 ± 0.53	4.09 ± 0.41 *	−2 (−8 to 9)	68	4.33 ± 0.69	4.26 ± 0.44	−2 (−10 to 8)	210	4.22 ± 0.51	4.10 ± 0.40 *	−4 (−11 to 4)
Ultrasound parameters	TAPSE (mm)	573	14 (12–16)	14 (12–16)	0 (0 to 0)	363	14 (12–17)	14 (12–16)	0 (0 to 0)	35	15 (12–17)	15 (12–17)	0 (−11 to 0)	175	14 (12–16)	15 (12–16)	0 (0 to 0)
	RVEDD (mm)	683	39 (33–45)	38 (33–44) *	0 (0 to 0)	432	39 (33–47)	39 (33–47)	0 (0 to 0) †	59	32 (26–38)	31 (26–36) *	0 (−5 to 0)	192	39 (34–43)	38 (34–43) *	0 (0 to 0)
	LVEDD (mm)	692	39 (35–45)	39 (35–45)	0 (0 to 0)	438	38 (33–44)	37 (33–44)	0 (0 to 0)	61	50 (44–55)	50 (43–55)	0 (0 to 2)	193	39 (36–42)	39 (36–43) *	0 (0 to 3)

Alt, glutamic pyruvic transaminase; Cr, Creatinine; DBP, diastolic blood pressure; HF, heart failure; HR, heart rate; LVEDD, left ventricular end-diastolic diameter; MAP, mean arterial pressure; NT-proBNP, N-terminal prohormone of brain natriuretic peptide; rhBNP, recombinant human brain natriuretic peptide; RVEDD, right ventricular end-diastolic diameter; SBP, systolic blood pressure; TAPSE, tricuspid annular plane systolic excursion; TBil, total bilirubin; UA, uric acid. *: *p* < 0.05 for comparison between pre- and post-rhBNP. **: *p* < 0.001 for comparison between pre- and post-rhBNP. †: *p* < 0.025 for comparison of Group 1 or Group 4 pulmonary hypertension patients with Group 2 pulmonary hypertension patients.

## Data Availability

Our data are unavailable due to privacy or ethical restrictions.
